# Multicentre, randomised controlled feasibility study to compare a 10-week physiotherapy programme using an interactive exercise training device to improve walking and balance, to usual care of children with cerebral palsy aged 4–18 years: the ACCEPT study protocol

**DOI:** 10.1136/bmjopen-2021-058916

**Published:** 2022-05-30

**Authors:** Rachel Rapson, Jonathan Marsden, Jos Latour, Wendy Ingram, Kara Nicola Stevens, Laura Cocking, Bernie Carter

**Affiliations:** 1 Physiotherapy, Children and Family Health Devon, Torbay and South Devon National Health Service Foundation Trust, Torquay, UK; 2 Faculty of Health and human Sciences, University of Plymouth, Plymouth, UK; 3 University of Plymouth Faculty of Health and Human Sciences, Plymouth, UK; 4 University of Plymouth School of Nursing and Midwifery, Plymouth, UK; 5 Peninsula Clinical Trials Unit, Plymouth University Peninsula Schools of Medicine and Dentistry, Plymouth, UK; 6 Medical Statistics, University of Plymouth, Plymouth, UK; 7 Peninsula Clinical Trials Unit at Plymouth University (PenCTU), University of Plymouth, Plymouth, UK; 8 Faculty of Health and Social Care, Edge Hill University, Ormskirk, UK

**Keywords:** Paediatric neurology, REHABILITATION MEDICINE, Developmental neurology & neurodisability

## Abstract

**Introduction:**

Children with cerebral palsy (CP) frequently undertake physiotherapy programmes to improve walking and balance. They often require adult support to exercise in a functional position. A novel interactive exercise trainer has been devised to enable children to exercise with against resistance in a functional position, but its efficacy has yet to be proved. A novel protocol has been developed to determine whether a randomised controlled trial (RCT) is feasible.

**Aim:**

To establish whether it is feasible to conduct an RCT to assess the effectiveness of a 10-week physiotherapy intervention using an interactive trainer in children with CP.

**Methods and analysis:**

This study is multicentre randomised controlled feasibility trial with an embedded qualitative study. Forty children with CP, Gross Motor Function Classification System (GMFCS) I–III will be recruited from community paediatric physiotherapy caseloads. Participants will be randomised to 10 weeks of training with the interactive training device or to usual physiotherapy care. The mediolateral motion of the centre of mass estimate and Paediatric Balance Scale will be explored as potential primary outcomes measures, tested at baseline, 10 weeks and follow-up at 20 weeks. The views of child participants, their parents and physiotherapists will be gained through e-diaries and qualitative interviews.

Feasibility will be determined by examining recruitment and retention rates, completeness of, adherence to the intervention, appropriateness of outcome measures and effectiveness of blinding. Results will be reported in accordance to Consolidated Standards of Reporting Trials (CONSORT) guidelines.

**Ethics and dissemination:**

Physiotherapists, children and parents have informed trial design and information leaflets. Results will be disseminated via publications, conferences and to families. This study has approval from North of Scotland Research Ethics Committee (20/NS/0018).

**Trial registration number:**

ISRCTN80878394.

**Strengths and limitations of this study**

- We use a mixed-methods approach to assess the feasibility of a proposed randomised controlled trial.
- This protocol tests the feasibility of two potential primary outcome measures.
- This study is not designed to determine differences in outcome measurements.

## Introduction

Cerebral palsy (CP) is a group of permanent disorders affecting the development of movement and posture that occurs in 2.1 per 1000 children worldwide.^1^ Difficulties with walking and balance are common and can limit participation in schooling and functional activities.^2–5^ There are multiple causes of walking difficulties in children with CP, including spasticity and weakness, which affects 80% of children.^6^ Additionally, children with CP often have poor balance, which further impacts on everyday functional tasks, such as dressing.^7^

Walking ability can be classified using the Gross Motor Function Classification system (GMFCS).^8^ Children with GMFCS I–III are the focus of the proposed study. Children with GMFCS classification I–II are able to walk functionally outdoors, while children with grade III GMFCS require walking aids.

**Figure 1:**
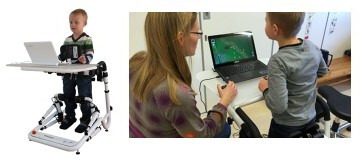
The Happy Rehab interactive exercise gaming device. Permission obtained from Innovaid.

Physiotherapists frequently prescribe exercise programmes for children with CP aimed at maintaining range of movement, strengthening weak muscles and developing balance skills. In many cases, the children find it hard to undertake exercises in functional positions such as standing, without support from an adult. The Happy Rehab (Innovaid, Denmark) interactive exercise trainer was developed (see figure 1) to help children exercise more independently in a functional supported standing position. It provides support around the hips and additional assistance and resistance via motors aligned to the ankle and knees. This allows the child to exercise muscles functionally in novel ranges, for example, strengthening the thigh muscles with the hip and knee in a straighter position. The games-based exercises may increase motivation and require the child to control the games by moving their weight side-to-side, forward and backward. It is proposed that this may improve balance during dynamic tasks such as walking.

A small scale study of the interactive trainer found marked improvements in walking, but had a number of limitations in terms of outcome measures used, lack of follow-up and control group.^9^ Therefore, evidence is still required to establish the efficacy of the equipment. Initially, a study is required to establish the feasibility of such a trial within a community physiotherapy service.

This study aims to establish whether it is feasible to conduct a randomised controlled trial (RCT) of this complex intervention, and to assess the acceptability of the interactive trainer and the trial protocol to physiotherapists, children and their families.

## Objectives

The objectives of the study are to:

1. Determine the feasibility of a definitive trial.
2. Determine the acceptability of the intervention.
3. Explore the views of a subgroup of study participants.

The trial objectives will be measured by the outcomes set out in table 1.

**Table 1:** Objectives of the feasibility study

Objective	Outcome
**Focus**	**Methods**
Feasibility of definitive trial	
Acceptability of the trial and intervention	Interviews of staff, parents and children
Can we recruit and retain participants?	No of participants eligible No recruited and randomised, date of recruitment recorded on study database Recruitment source No of withdrawals. No of participants lost to follow-up.
Effectiveness and acceptability of randomisation	Comparison of participant characteristics: severity, distribution of motor impairment, associated impairments at baseline Interviews
Effectiveness of concealment of allocation up to week 10	No of times chief investigator correctly guessed treatment allocation
Concurrence with other surgical and medical interventions	No of operations or procedures that target balance and walking during the intervention and follow-up period.
Change in clinical outcome measures	Change in assessment scores of outcome measures
Assess appropriateness of outcome measures	No and percentage of outcome measures completed at each time point Interviews
Feasibility of Intervention	
Adherence to treatment	Diary data frequency and duration of training
Acceptability of treatment intervention	Incidence of breakdown of equipment No of times participants were unable to access equipment Participant view on acceptability of interventions by interview
Cost of intervention and support needed to use it	Local physiotherapist record of staff time and grade used to support intervention. Travel costs of staff and families. No and cost of repairs
Safety of intervention	No and type of SAE and AE
Acceptability of participation	
Acceptability of participation	Themes identified from interviews/photos

AE, adverse event; SAE, serious adverse event.

## Methods and analysis

### Trial design and setting

The research question is as follows: Is it feasible to conduct a multicentre randomised control trial of a physiotherapy programme using an interactive exercise trainer to improve balance in ambulant children with CP? This trial is a single-blinded; multicentre feasibility RCT with embedded qualitative study. Community paediatric physiotherapists working at Child Development Centres (CDCs) will recruit children from their caseloads. The study will be conducted between 9 February 2021 and 1 August 2022. Participants will be randomly allocated to training with the Happy Rehab device, or to the control group of usual physiotherapy care. Both groups will carry out 10 weeks training at home, the clinic or their school. The study will compare the intensity of training in different settings. Qualitative semistructured interviews with a subgroup of physiotherapists, parents and children will take place to explore their experiences of taking part; interviews will take place in the clinic or child’s home.

The research question can be framed in the following way:

P Population—Children with CP aged 4–18 years.

I Intervention—A programme of physiotherapy using the interactive training device.

C Comparison group—Usual care.

O Outcome of interest—Feasibility of the trial and intervention.

T Time—Training three times per week for 10 weeks, plus follow-up at week 10 and week 20.

**Figure 2:**
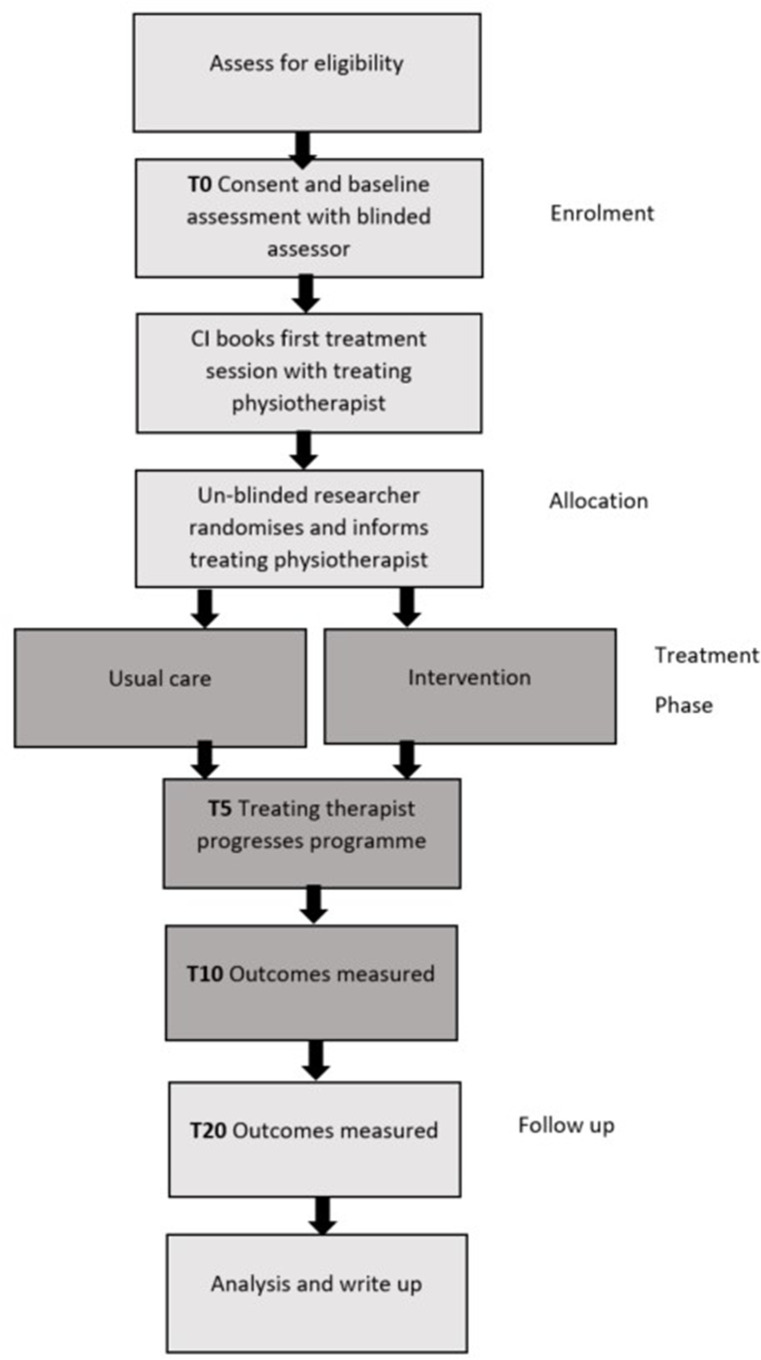
Trial flow diagram. CI, chief investigator; T, time in weeks.

The trial flow chart is shown in figure 2.

### Participants

The eligibility criteria for participants are shown in box 1.

Box 1 **Eligibility criteria**

**Inclusion criteria**

- Diagnosis of CP GMFCS I–III.
- Aged 4–18 years.
- Leg weakness (≤4/5 on the Medical Research Council (MRC) muscle strength rating scale) in at least 1one muscle group.
- Leg hypertonia (≥1 on the Tardieu scale fast stretch) in at least 1one muscle group.
- Ability to interact with a computer game using a mouse or joystick.

**Exclusion criteria**

- Selective dorsal rhizotomy or multilevel orthopaedic surgery within the last 12 months.
- Soft tissue surgery in lower limbs in last 6 months.
- Botulinum toxin injections in the lower limbs within previous 3 months.
- Training with the Happy Rehab in the last 4 months.

### Intervention

Four devices will be available and situated in special schools, CDCsor the child’s home. The child’s physiotherapist will be trained to set up the device targeting exercises to improve range of movement, contracture and muscle weakness. This may include active-assisted hip, knee or ankle movements within specified ranges of movement or side-to-side and forward and back weight transfer. The treating physiotherapist will personalise the exercise programme based on a standardised assessment, including a discussion with the child and their guardian about their goals and aims of any intervention.

The child will use a pseudonym of their choice to log onto the games, and to maintain confidentiality. Children will play the games within the mid-range of muscle length to begin with so that the games are difficult but achievable. This will aid motivation and adherence. After 5 weeks, the child’s physiotherapist will progress the games by requiring the muscles to work in the inner and outer ranges of movement and/or against increased resistance. Training will build up to 20 min per day, 3 days a week over a 2-week period, with progression to a 30 min programme per day, 3 days a week after 5 weeks. The child will train with supervision from by the child’s therapist, teaching assistant, parent or carer. The children will follow a series of games following a 2 min warm up of continuous passive movement. The interactive trainer records the training session (duration, games performed games outcomes) to provide a description of the parameters of training.

The control group will receive a usual care physiotherapy programme individualised for each child lasting 20–30 min.

Collaborative goal setting combined with an e-diary will allow the children and their parents/carers from both arms of the study to monitor progress over time and record their satisfaction with their exercise programme. The research team will assess fidelity through e-diaries, recording of exercise parameters via the interactive trainer and by observing ten exercise sessions and completing a fidelity checklist, to ensure the intervention follows protocol.

### Study procedures

#### Site setup

Recruitment will take place sequentially in each CDC area in order to ensure that the limited number of training devices are issued in the most efficient way. In preparation for recruitment, the research team will visit each site to familiarise physiotherapists with the eligibility criteria and trial procedures.

#### Recruitment

The physiotherapist will approach children and families on their caseload and give an information pack. Adverts for the study will be placed in clinic rooms, and on parent forums and social media. Potential participants who respond to the invitation will be screened for suitability using a telephone questionnaire to check diagnosis, age, GMFCS level and ability to play a game using a mouse or joystick. Eligible families will be approached for consent to be recruited to the qualitative study at baseline using a purposive sampling framework.

Potential participants who do not wish to take part in the study or withdraw from the study will be invited to undertake a short (less than 5 min) telephone interview to help understand any barriers and facilitators to participating in the trial, to aid in future recruitment. A separate information sheet will be available for these interviews.

### Data collection

#### Baseline visit

Written informed consent and assent will be recorded prior to the child and parent undertaking the first baseline measurement session. The following data will be collected at the first visit:

- GMFCS level.
- Date of birth.
- Medical and surgical history.
- Height, weight, pelvic depth.
- Frequency and location of usual physiotherapy.
- Other sports and social activities.

### Outcome measures

The following assessments will be carried out at weeks 0 and 10, and those indicated with * at 20 weeks follow-up. The physical assessments will take 70 min, followed by goal setting. Qualitative semistructured interviews will take place after completion of the 10-week training.

#### Primary outcomes

*Medio-lateral motion of the centre of mass estimate.^10 11^
*Paediatric Balance Scale.^12^

#### Secondary outcomes

*Walking kinematics*.
*Muscle strength of quadriceps, hamstrings, and gastrocnemius and hip abductors using a hand held dynamometer (three measurements).
*Passive range of movement and modified Tardieu scale^13^ of quadriceps, hamstrings, gastrocnemius and hip adductors using goniometer (three measurements).
- Canadian Occupational Performance Measure.^14^
- Child Health Utility instrument -CHU-9D—Paediatric Quality of Life measure.^15^

#### Blinding

This will be a single-blinded RCT. The assessor will be blinded to allocation while carrying out the assessments at baseline and week 10. It will not be possible to for the assessor to remain blinded to group allocation for the 12 participants taking part in qualitative interviews occurring at week 11. However, the assessor will remain blinded to group allocation at week 20 for the remaining participants who are not undertaking the interviews.

#### Randomisation

Participants will be randomly allocated at a ratio of 1:1 and will be minimised by age (above or below 9 years) and by GMFCS level (level I and II vs level III). This is because acquisition of gross motor ability peaks by age 9, and children above that age plateau or may decline in motor skills.^16^ The minimisation sequence and randomised allocations will be computer-generated in conjunction with an independent statistician. The blinded assessor will enter the details required for randomisation into the study website, book the participant’s first appointment with the treating therapist.

An email will be generated by the study website to inform the local treating physiotherapist of the participant’s allocated group. The treating physiotherapist will reveal group allocation to the participant at the first session. An unblinded researcher will arrange for the interactive trainer to be transported to the site where the child usually does their physiotherapy for example, school, CDC, home.

#### Qualitative assessment

This qualitative study uses novel ways of data collection with the children including semistructured e-diaries using electronic tablet devices and photo-elicitation interviews. Semistructured interviews will be undertaken with parents/carers and physiotherapists. Triangulation of the e-diaries and interviews will be used to provide credibility, ensuring that the understanding of the full scope of the experiences related to participating in the trial is as complete as possible from the perspectives of the children, parents and physiotherapists. Twelve parent–child dyads will be recruited (30% of the total sample of the feasibility study). Four physiotherapists, who have delivered the intervention and control treatments in different settings, will be interviewed. Sampling of up to eight parents who declined or withdrew their child from the study will be undertaken.

### Statistical analysis plan

A statistical analysis plan (SAP) will be drafted prior to the final database lock; the SAP will be agreed with the trial steering committee (TSC) in the absence of a data monitoring committee. A CONSORT diagram will be used to present descriptive data on screening, enrolment, intervention allocation, follow-up and assessment.

Completion rates of the intervention and outcomes collected at each time point will be reported with confidence intervals. All analyses and data summaries will be conducted on the intention-to-treat population, defined as all participants randomised regardless of non-compliance with the protocol or withdrawal from the study. Participants will be analysed according to the intervention they received.

The baseline characteristics of those lost to follow-up will be compared with those who complete the trial in order to identify any potential bias.

#### Proposed primary and secondary outcome analysis

The planned primary and secondary outcome measures will be reported at each time point using descriptive statistics. As this is a feasibility trial, it is not appropriate to perform a hypothesis test between-group treatment effects.^17^ Instead, the difference between allocated groups of the follow-up minus baseline score will be estimated with confidence intervals.

A sample size estimate for a definitive trial will be undertaken for the proposed primary outcome. Estimation of the SD, correlation between baseline and follow-up measures and a clinically meaningful difference will be used in the power calculation.

#### Progression criteria

This is determined in advance of recruitment will include minimum recruitment and retention rates (~70%) and a 90% completion rate of outcome measures. Failure to achieve these will indicate that a full trial is not feasible unless our qualitative study indicates clear means by which the rates may be improved. A recommendation list will be generated to enable refinement of the subsequent RCT protocol.

#### Qualitative analysis and data synthesis

Qualitative data will be analysed using thematic analysis. Results from all aspects of the quantitative and qualitative data will be triangulated and synthesised and will be used to determine the suitability of the protocol for incorporation into the main RCT.

### Public and patient involvement and engagement

Families and physiotherapists have been consulted on trial design, with a particular focus on the two assessment visits. Documents including the protocol, adverts and patient information sheets were reviewed by an expert parent and a teenager with CP, and altered to make the information more accessible. Emerging themes from the qualitative analysis will be checked and informed by an invited group of children, parents and physiotherapists with relevant experience.

### Data collection and management

Trial data collected will be recorded on a paper copy of a trial-specific case report form (CRF) and will be considered source data. The blinded assessor will complete the CRFs for all participants. Completeness of data will be maximised by checking all forms at each assessment to ensure there are no missing items. Automatically generated prompts will be sent by email to encourage the participants to return their diaries, should they fail to do so within 2 weeks of the due date. Double-entered data will be compared for discrepancies using a stored procedure, and discrepant data will be verified using the original paper data forms. Before database lock, a proportion of original paper records will be checked against the database to ensure accuracy of the final dataset.

Confidentiality will be maintained by allocating a participant number to all CRFs and keeping the securely codes stored separately. Audiorecorded interviews will be transcribed and anonymised as soon as practicable. Original recordings will be held securely as an encrypted file on the University of Plymouth server, until completion of the qualitative data analysis process, then deleted.

Data will be collected and stored in accordance with the Data Protection Act 1998/General Data Protection Regulation 2018. Data generated from this trial will be available for inspection on request by the participating research team, University of Plymouth representatives, the REC, local R&D Departments and the regulatory authorities.

### Sample size

As this study is a feasibility trial, it is not appropriate to use a sample size calculation based on considerations of power for detecting between group differences.^17^ The feasibility aims are to provide robust estimates of recruitment rate and follow-up as well as estimates of the variability of the outcome measures, which will in turn inform sample size calculations for a full RCT.

A sample size of 40 participants will allow the overall recruitment rate to be estimated. It is anticipated that follow-up of a minimum of 12 participants in each of the intervention and usual care groups would provide sufficient data to inform indicative sample size calculations for the definitive main trial. An estimated recruitment rate of three to four children per month over a 12-month period has been calculated based on population and previous experience.

### Adverse events

The risks of taking part in this trial have been assessed to be low. Three adverse events (AEs) that require reporting include aches and pains in the leg muscles following training that last over 1 hour or require pain relief, injury related to the training and fatigue lasting more than 1 day following training. AEs will be recorded via the online diary. Recorded AEs and serious AEs will be presented to the monthly trial management group meeting for review.

### Roles and responsibilities

The roles and responsibilities are shown in table 2. The trial management group TMG consists of R Rapson’s supervisory team Professor Jos Latour, Professor Bernie Carter, Professor Jonathan Marsden, Rachel Rapson, CTU Trial Manager (Dr Wendy Ingram), CTU Data Manager (Laura Cocking) and trial statistician (Dr Kara Stevens). The TSC consists of an independent chairperson, statistician, PPI representatives, sponsors representative and local research and development manager.

**Table 2:** Roles and responsibilities of protocol contributors

Chief investigator	Rachel Rapson Peninsula Allied Health Centre University of Plymouth PL6 8BH rachel.rapson@plymouth.ac.uk
Trial coordinator	Jonathan Marsden Peninsula Allied Health Centre University of Plymouth PL6 8BH Jonathan.marsden@plymouth.ac.uk
Sponsor	Sarah.C.Jones University Sponsor Representative Research and innovation Drake Circus Plymouth PL6 8AA Plymouth.sponsor@plymouth.ac.uk
Funder(s)	National Institute of Health Research
Clinical trials unit	Wendy Ingram Clinical Trials Manager Peninsula Clinical Trials Unit University of Plymouth Wendy.ingram@plymouth.ac.uk
Data manager	Laura Cocking Senior Data Manager Peninsula Clinical Trials Unit University of Plymouth Laura.cocking@plymouth.ac.uk
Trial statistician	Kara Stevens Research Fellow in Medical Statistics, University of Plymouth Kara.stevens@plymouth.ac.uk

### Ethics and dissemination

The child’s assent and parental consent for their child’s participation will be sought at the start of the study. Rests will be offered during the measurement sessions, which will be conducted at the child’s pace. The child and family’s involvement is voluntary and they will be reminded that they can refuse any part of the study, or withdraw at any time without consequence to their treatment. This study has approval from North of Scotland Research Ethics Committee (20/NS/0018) and the respective NHS Research and Development departments.

The University of Plymouth research team will own the data arising from the trial. On completion of the trial, the data will be analysed and tabulated and a final trial report prepared. Study findings will be published in peer reviewed academic journals and presented at national and international conferences. National Institute for Health and Care Research (NIHR) funding will be acknowledged within the publications. The outcomes of the trial will be shared with participants using a lay summary. Anonymised participant level data set will be available 1 year after the end of the trial via the Rehabilitation Research Group (University of Plymouth) website.

## Discussion

This study sets out to explore the feasibility of conducting a trial using a complex intervention in a variety of community settings. Using the proposed protocol, we will explore barriers and facilitators to running the trial. The protocol sets out a model of loaning the training device for an intensive ten-week intervention. We will be collecting initial data to indicate cost of the device, transport, repairs and maintenance. We anticipate that the logistics of transporting the devices within the community may prove difficult using existing infrastructures. We will be able to test these procedures to gain realistic timescales for a full trial.

We plan to include children with a range of cognitive, sensory and motor skills and we will examine if our primary outcome will capture change across all participants. By engaging in qualitative interviews, we will be able to determine the children and their parents’ experiences of and perspectives on both the intervention and proposed outcomes, gaining important information whether these were acceptable or if outcomes were too difficult or took long. We will be able to examine their perspectives on any impact that the location (school, home or clinics) has on participation, as well the wider impact on their levels of participation and ability to manage their condition.

The main limitation of the trial is the lack of power to determine a significant difference in outcome measures. However, this feasibility study is an important step towards designing a full trial to test the efficacy and economic benefit of using a novel interactive exercise trainer to improve walking and balance in children with CP.

## Supplementary Material

Reviewer comments
