# Supplementary material for: Multicentre, randomised controlled feasibility study to compare a 10-week physiotherapy programme using an interactive exercise training device to improve walking and balance, to usual care of children with cerebral palsy aged 4–18 years: the ACCEPT study protocol
Source: BMJ Open. 2022 May 30;12(5):e058916. doi: 10.1136/bmjopen-2021-058916 (PMC9152930; doi:10.1136/bmjopen-2021-058916)
Supplement: Reviewer comments [file bmjopen-2021-058916.reviewer_comments.pdf]

## PEER REVIEW HISTORY

BMJ Open publishes all reviews undertaken for accepted manuscripts. Reviewers are asked to complete a checklist review form (**http://bmjopen.bmj.com/site/about/resources/checklist.pdf**) and are provided with free text boxes to elaborate on their assessment. These free text comments are reproduced below.

## ARTICLE DETAILS

| | |
|---|---|
| **TITLE (PROVISIONAL)** | A multicentre, randomised controlled feasibility study to compare a 10-week physiotherapy programme using an interactive exercise training device to improve walking and balance, to usual care of children with cerebral palsy aged 4-18 years. : The ACCEPT study protocol. |
| **AUTHORS** | Rapson, Rachel; Marsden, Jonathan; Latour, Jos; Ingram, Wendy; Stevens, Kara; Cocking, Laura; Carter, Bernie |

This article was not externally reviewed at BMJ Open. Protocols that have been independently assessed prior to submission to BMJ Open are usually fast-tracked to publication on the grounds that further substantial changes will not be possible. This independent assessment will usually be external, independent review for both a substantial grant award from a non-commercial or government funder and ethics approval.
